# Common sleep data pipeline for combined data sets

**DOI:** 10.1371/journal.pone.0307202

**Published:** 2024-08-06

**Authors:** Jesper Strøm, Andreas Larsen Engholm, Kristian Peter Lorenzen, Kaare B. Mikkelsen

**Affiliations:** Department of Electrical and Computer Engineering, Aarhus University, Aarhus N, Denmark; U of M: University of Minnesota, UNITED STATES OF AMERICA

## Abstract

Over the past few years, sleep research has shown impressive performance of deep neural networks in the area of automatic sleep-staging. Recent studies have demonstrated the necessity of combining multiple data sets to obtain sufficiently generalizing results. However, working with large amounts of sleep data can be challenging, both from a hardware perspective and because of the different preprocessing steps necessary for distinct data sources. Here we review the possible obstacles and present an open-source pipeline for automatic data loading. Our solution includes both a standardized data store as well as a ‘data serving’ portion which can be used to train neural networks on the standardized data, allowing for different configuration options for different studies and machine learning designs. The pipeline, including implementation, is made public to ensure better and more reproducible sleep research.

## Introduction

Reproducibility in science is important, which necessitates access to common benchmark data sets. However, science does not truly become reproducible until this access includes standardized, accepted methods for handling and processing this data, where it is needed.

The move towards reproducibility in sleep research has been growing stronger over a long time, with notable developments being the creation of the National Sleep Research Resource [1] and the growing number of open and pretrained sleep analysis models.

Most recently, several studies have highlighted differences between data sets and more generally between sleep data and sleep scorings originating at different sleep clinics [2–5]. This shows that high quality sleep studies, at least under the current paradigm of the American Academy of Sleep Medicine’s guidelines [6], should strive to include data from multiple different sleep clinics; otherwise the results are likely to have uncontrolled biases depending on the sleep clinics represented.

Naturally, such a development puts further pressure on the researchers, and creates a need for additional tools to work with such large and seemingly ever expanding benchmark data sets.

Therefore, in this paper, we present an open source pipeline for working with open sleep data sets. Starting from reasonable design constraints aimed at broad usability, we identify sound solutions to these which maximize performance and reusability. We then implement these in an analysis pipeline which can be freely downloaded from our GitHub repository (see below). An overview of the datasets used for this study and their detailed information can be found in Table 1.

**Table 1:** Overview of the 21 datasets used in the pipeline.

Dataset	Subjects	Records	Length (30s epochs)	Size in GB^1^	Training^2^ / Hold-Out^3^
ABC	49	132	133 101	39	Training
CCSHS	515	515	691 402	119	Training
CFS	730	730	866 222	149	Training
CHAT	1 232	1 639	1 958 341	673	Training
DCSM	255	255	578 939	67	Training
HPAP	247	247	231 436	62	Training
MESA	2 055	2 055	2 606 541	373	Training
MROS	2 903	3 921	5 398 538	743	Training
PHYS	919	919	847 346	170	Training
SEDF-SC	76	145	394 214	34	Training
SEDF-ST	21	36	34 857	3	Training
SHHS	5 797	8 444	9 055 971	1 100	Training
SOF	453	453	541 696	93	Training
ISRUC-SG1	91	91	79 600	19	Hold-Out
ISRUC-SG2	6	6	5 141	1.2	Hold-Out
ISRUC-SG3	10	10	8 589	2	Hold-Out
MASS-C1	40	40	42 911	25	Hold-Out
MASS-C3	61	61	61 853	35	Hold-Out
SVUH	25	25	20 789	2.4	Hold-Out
DOD-H	25	25	24 665	7	Hold-Out
DOD-O	56	56	54 197	2.2	Hold-Out
**Total**	15566	19805	23636349	3719	

^1^Sizes refer to the preprocessed datasets.

^2^Used for both training, validation and test.

^3^Purely used for testing.

In our implementation, we have assumed that open source researchers work in Python (version ≥ 3.9), and have favoured, in our own tests, the Pytorch deep learning framework, which we consider the dominant framework in academia. In the present work, we have focused on data from head mounted electrodes (EEG and EOG), however, there is no reason why the pipeline could not easily be extended to other modalities, such as movement or breathing sensors.

We name our proposed solution the ‘Common Sleep Data Pipeline’, and will refer to it interchangeably as either ‘CSDP’ or simply ‘the pipeline’.

### Repository

All the code is publicly available at https://github.com/jesperstroem/CSDP. A demonstration of the pipeline is available at https://github.com/jesperstroem/CSDP-demonstration. Results from the demonstration will be presented in this article.

## Related work

While the multiple works have highlighted the need for combining multiple data sets [2–5], none of these have directly addressed the issues of working with such large volumes of data. The focus have been on specialized pipelines for specific purposes. As such, reusing the existing code and data to answer new research questions can be difficult. From this follows that research becomes harder to compare because the experimental setups all vary in different ways. To combat this, we opted to create a standardized solution, where the ambition is to make the results reproducible, and the code reusable and extendable.

Recently, Sveinbjarnarson et al. [7] has described the design of a sleep data pipeline for multiple data sources. This work also describes the challenges in working with multiple data sources and discusses solutions to them. Their design describes a setup and formula for how to transform multiple heterogeneous datasets into a single common homogenous database, comparable to our approach on breaking down the different operations needed to preprocess a dataset for sleep research. The main difference between this study and the present work is that while Sveinbjarnarson et al. describe a fairly high level design, which must subsequently be translated into an implementation, we here present both describe a design and implement it. Additionally, Sveinbjarnarson et al. appear mostly interested in the database management part, while the present study also proposes an implementation of a flexible sleep-staging pipeline with data loading capabilities.

## Materials and methods

To ensure the highest degree of generalization and applicability, we have designed our implementation relative to both best practices in software development as well as common, standard use cases, as they are described today in the sleep analysis literature. In this section we will go through the different design constraints.

### Template method pattern for data transformation

Transforming a sleep data set involves a lot of common steps which happen for every data set and always in the same order. This can be thought of as a recipe. However, the implementation of some steps may vary between data sets, and must therefore be customizable for the individual data set. Therefore, we chose to base the design on the Template Method design pattern [8]. In this software pattern, an abstract base class defines a template method, which describes the order of a collection of operations, but not how they are implemented. Concrete implementations that inherit from the base class define that logic but keep the interface, order of operations and any default implementations. This enables writing new implementations when a new data set is added, while not having to re-implement trivial steps or the order in which they happen.

### Common data store

As discussed above, our solution needs to bring a heterogeneous collection of data sets into a common format which is both simple enough to realistically fit a large number of data sets, and complex enough to support many different analysis outputs. Concretely, it must be reasonably compatible with many different EEG montages, it must support widely different data set sizes and it must preserve the night/subject/data set hierarchical structure of the original data.

To ensure that we did not restrict ourselves to a particularly homogeneous subset of open sleep data sets, we decided to include all 21 data sets used by Perslev et al. 2021 [5], see Table 1. We also used the same distribution of training and hold-out sets.

### Computing environment

The best solution is likely to depend on the hardware on which the code will run. To avoid designing to our local setup, we have focused on the ‘LUMI’ (‘Large Unified Modern Infrastructure’, [9]) super computer. The LUMI consortium strives to deliver modern computing resources, and by tailoring our implementation to that environment, we believe we will increase the likelihood of a generally useful software solution.

### Data loading

As our focus is on ‘big data sleep research’, we have looked for solutions which are flexible towards data sizes—even using the cluster nodes of LUMI-G with over 500 GB of RAM, it is possible to run out of memory. It is also important to consider bottlenecks in on-the-fly data preprocessing and how to possibly parallelize within and between computing nodes.

### Demonstration

To demonstrate the utility of the pipeline, we fed the previously mentioned 21 different data sets (Table 1) through the pipeline, and into two different, state-of-the-art sleep scoring neural networks: U-Sleep [5] and L-SeqSleepNet [10]. These two networks were chosen due to being new, high performing, and requiring very different input formats (raw, downsampled data vs. spectrograms). For reproducibility, we have implemented both networks in Pytorch Lightning [11]. These implementations are also available in the above linked repository.

## Results

**Fig 1:**
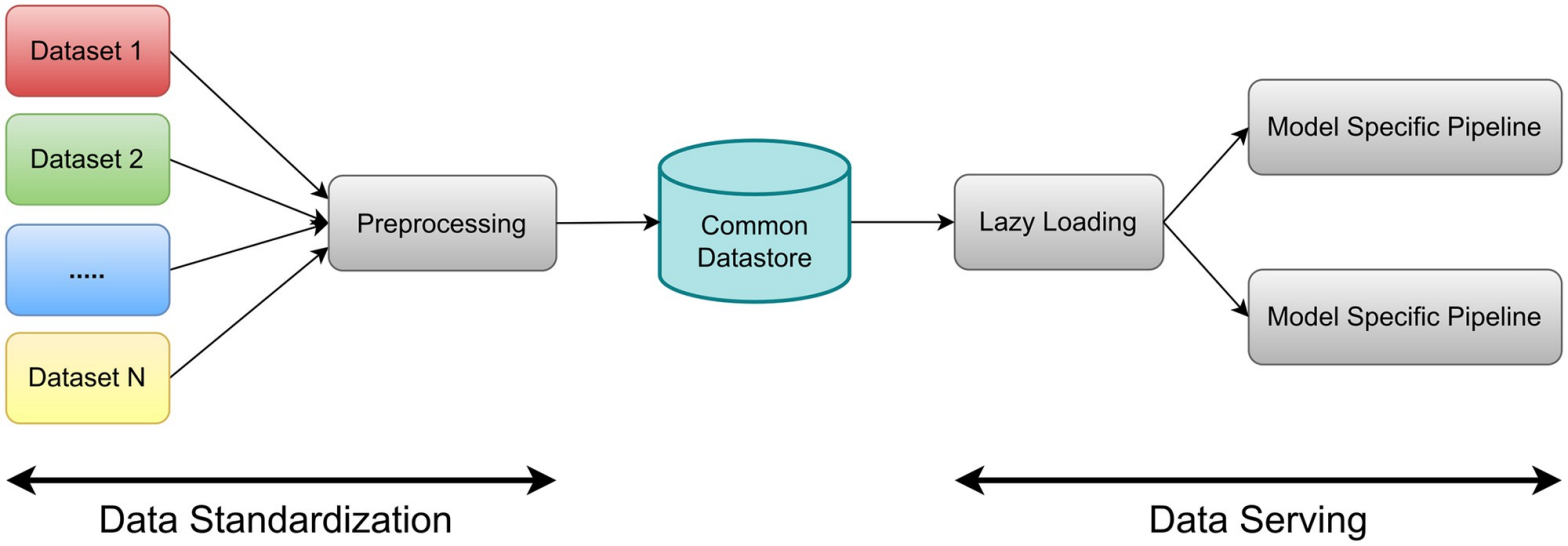
Overview of the total pipeline. The pipeline is separated in two: Data Standardization and Data Serving, with the common datastore connecting them. Lazy Loading refers to loading the preprocessed data from disk when it is needed during training.

We find that our proposed solution is best described as a combination of two parts: a data standardization portion culminating in a so-called ‘common data store’ and a subsequent data serving portion (see Fig 1). In this design, the data in the common data store tends to be less memory intensive than the original data sets, and we only have to perform the data standardization once. As is discussed below, the transformations performed in the second part of the pipeline (‘data serving’ in Fig 1) largely depend on the type of data augmention used and the requirements of the specific machine learning model under investigation.

We shall describe the two parts of the pipeline separately.

### Data standardization

#### Standardized format

We decided to rename all electrode positions based on the ‘10–10’ electrode positioning system [12], as that is the most general, standardized electrode positioning scheme, making it feasible to compare very different data sets. After renaming, all data was normalized to have zero mean and an interquartile range (IQR) of 1, and was resampled to 128 Hz. This sample rate was chosen due to being the highest of the sample rates used in the two reference works [5, 10], but could easily be changed to suit different needs.

As different data sets have different noise profiles, a simple approach to handling artefacts was chosen. In the current implementation we clip each channel to not exceed 20 times the IQR on a per-record and per-channel basis. This scheme is chosen based on the same methods used by Perslev et al. 2021 [5] because of it’s state-of-the-art results. Additionally, the pipeline supports bandpass filtering at user-defined cut-off frequencies in order to remove linear trends and high-frequency noise. More elaborate schemes can easily be implemented by modifying the relevant portion of the preprocessing template, see below.

As we utilize the 10–10 system, we do not consider any sensor modalities that are not in that standard. This means that information like body temperature or heart rate is not included in the data. It also does not include event-based data like unusual movements during sleep. However, due to the flexibility of the design, this can easily be included with small extensions to the code base, which will happen in a future version of the pipeline.

To reduce storage requirements, the user should define exactly which data channels are to be included in the common data store—all else will be discarded.

#### Structure of the common data store

After standardisation, recordings still need to be stored in a fashion which is both efficient during reading as well as flexible for different analyses. Addressing the latter part first, we have taken inspiration from the Brain Imaging Data Structure (BIDS) standard [13], which partitions data into the following hierarchy: ‘data set’ / ‘subject’ / ‘session’, where ‘session’ in our case corresponds to a night’s recording. Adopting this convention makes it easier to match the data storage with most common modes of analysis.

Following this decision, we need a file format which allows reading data according to this hierarchy:

#### File format

It is advantageous to pack standardized recordings in larger files containing multiple nights of data. The primary reason for this is the file systems used in high performance clusters, such as the LUMI cluster, have high overheads for individual file storage, and bundling of data sets are always encouraged.

We considered the most promising open file formats for aggregate data storage to be HDF5, Parquet and the Pickle format. In Table 2 is shown memory overhead and time delay in reading from the three file formats, extracting a single EEG channel from a single record from the CFS dataset (see Table 1). Memorywise, pickle shows poor results as it loads the entire file into memory, while HDF5 and Parquet loads smaller chunks of data. Concerning reading time, HDF5 is much faster than both Pickle and Parquet, by up to a factor of 10. Even though Parquet has the advantage of a very low peak memory, we consider reading time to be the most important metric. This is because the bottleneck of the machine learning system will be how fast data can be loaded into memory duing training. This reasoning explains our choice of HDF5 as the file format. The supporting data can be found in S1 and S2 Files. In our solution, each data set is saved into its own HDF5 file, using the subject / session structure as mentioned above. We believe this is the correct scale at which to bundle the data, to facilitate extension of the data store and easily sharing data sets between projects. Since HDF5 has the property that subfiles inside it can easily be extracted separately, there are few, if any, downsides to this convention.

**Table 2:** Analysis of Parquet, HDF5 and Pickle file formats, showing file size together with averages of peak memory and load time. File sizes were measured once as the amount of space used on the system disk. Memory and load time experiments were repeated 100 times for each file format and the mean is reported in the table. Peak memory showed negligible variance. The files all contained the same data from CFS record ‘cfs-visit5–800002’.

File Format	File Size (MB)	Peak Memory (MB)	Load Time (ms)
HDF5	883.9	147.3	49.7±16.1
Parquet	882.7	0.002	271.2±7.0
Pickle	883.9	883.9	290.8±22.1

#### Implementation

Using the template method mentioned above, we have designed a pipeline consisting of 9 steps, each of which can be exchanged independently, described in Table 3. As will be described below, for most data sets only steps 1–3 need to be re-implemented.

**Table 3:** Description of actions in the template method pattern adaption for the data standardization procedure, shown in order of operation.

#	Action	Description
1	List records	Lists all the filepaths of records in the dataset.
2	Read PSG	For every record, unwrap the data and labels as a PSG.
3	Map channels	Map the channel names to the 5 percent reference system.
4	Filter channels	Band-pass filter channels at user-defined cut-off frequencies
5	Scale channels	Scale values of every channel to a common reference point.
6	Clip channels	Clip every channel so that outliers are removed.
7	Resample channels	Resample every channel to 128 Hz.
8	Map labels	Map every label value to its corresponding AASM value.
9	Persist record^1^	Persist the psg record as an hdf5 file in the datastore.

^1^*Persist* refers to saving data for future access, in a manner where it outlives the process that created it.

**Fig 2:**
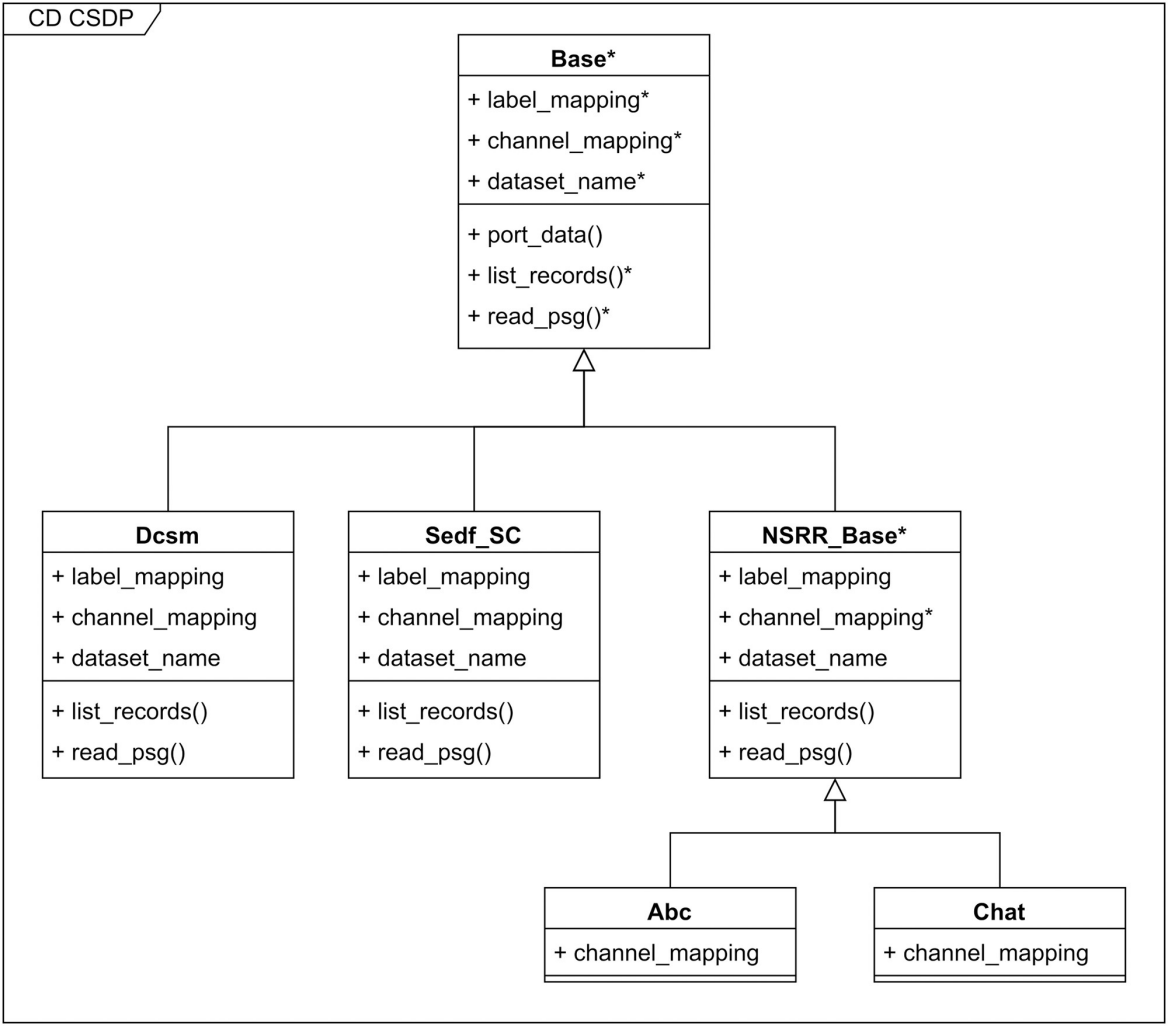
Class diagram depicting the inheritance hierarchy in CSDP. The arrows point towards the parent class, which the child class inherits its functionality from. Abstract classes, abstract functions and abstract properties are shown with *.

More specifically, Fig 2 shows an example of the class diagram, visualizing how specific data sets are incorporated into the pipeline. In our implementation the template method is called *port_data*, defining a recipe function to convert data into the common database. At the top is the abstract base class, which defines *port_data* and has 5 abstract members, implemented by the subclasses. Here, *label_mapping* defines how the labels translate to the AASM standard, the *channel_mapping* defines how the names of the channels translates to the 5 percent electrode standard. The base class also contains 2 abstract functions—*list_records* which needs to return a list of file paths for the records of the entire data set. Finally, *read_psg* needs to return the data and labels for each of the records previously listed.

Also shown in Fig 2, we created another abstract base class for all of the data sets originating from the National Sleep Research Resource (NSRR) [1], since those all have the same folder and file structure. On the figure this abstract base class is called “NSRR_Base”. This class defines everything but the channel mapping, which is almost always different from one data set to the other. Going forward, this codebase can be extended with new data sets by either creating new base classes or creating new concrete implementations of the existing base classes. For example, when NSRR releases a new dataset, the only new code that needs to be written is the channel mapping.

### Data serving

After data has been standardized and saved into HDF5 files, the last step is to load it back into the machine learning framework of choice. We have focused on accommodating the Pytorch framework, as we consider this the most commonly used deep learning framework in academia.

For very large data sets, especially when data augmentation is also considered, the best approach is to simply draw raw EEG sequences randomly. This is flexible in terms of distributed learning across multiple nodes, and allows evaluating the model more often than if all training data had to be processed in each iteration. This also removes the need to shuffle data before training, which would cost extra time and processing power.

**Fig 3:**
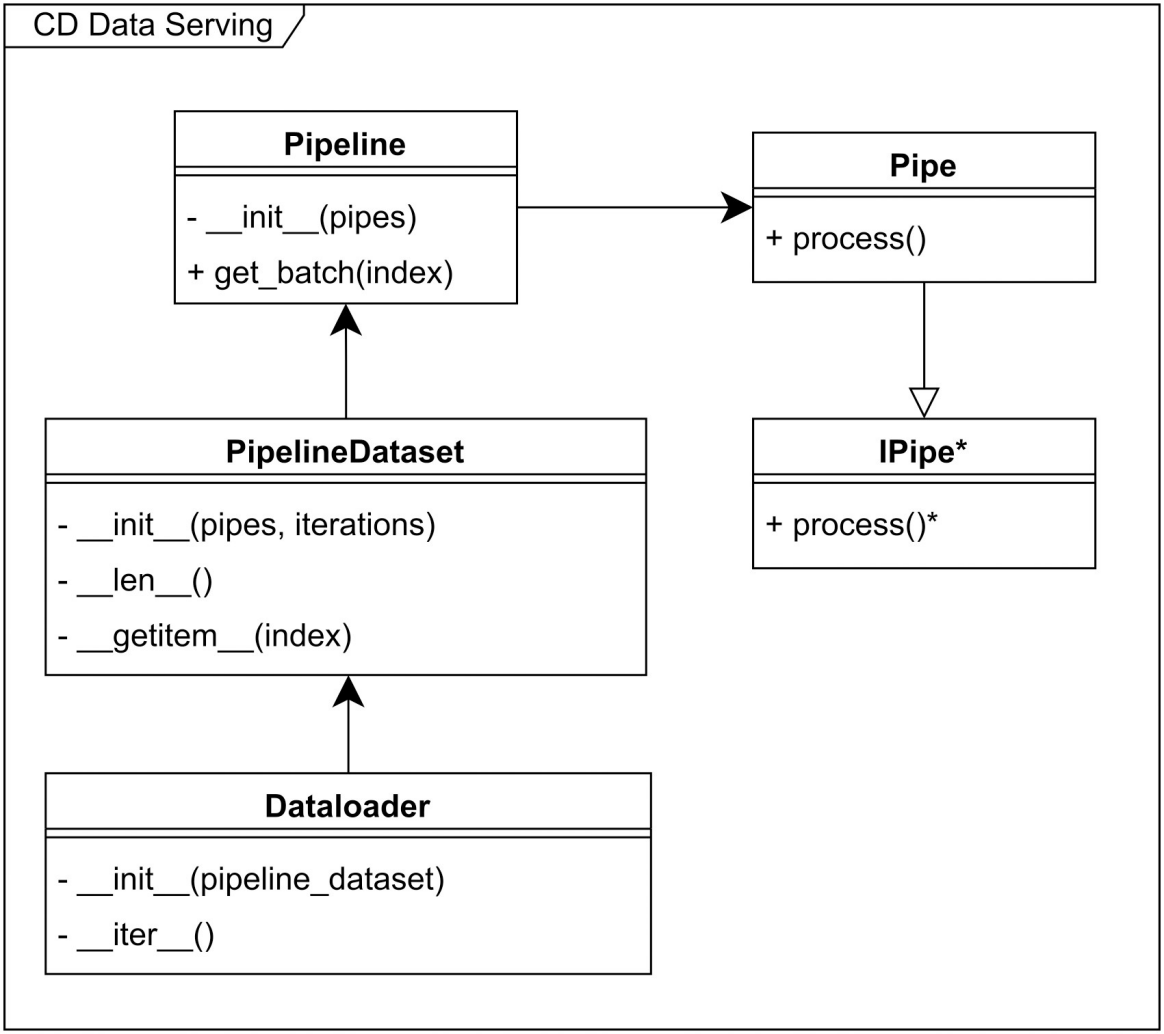
Class diagram depicting the dataserving part of the pipeline. A generic Pipe class is used. Abstract classes, abstract functions and abstract properties are shown with *.

**Fig 4:**
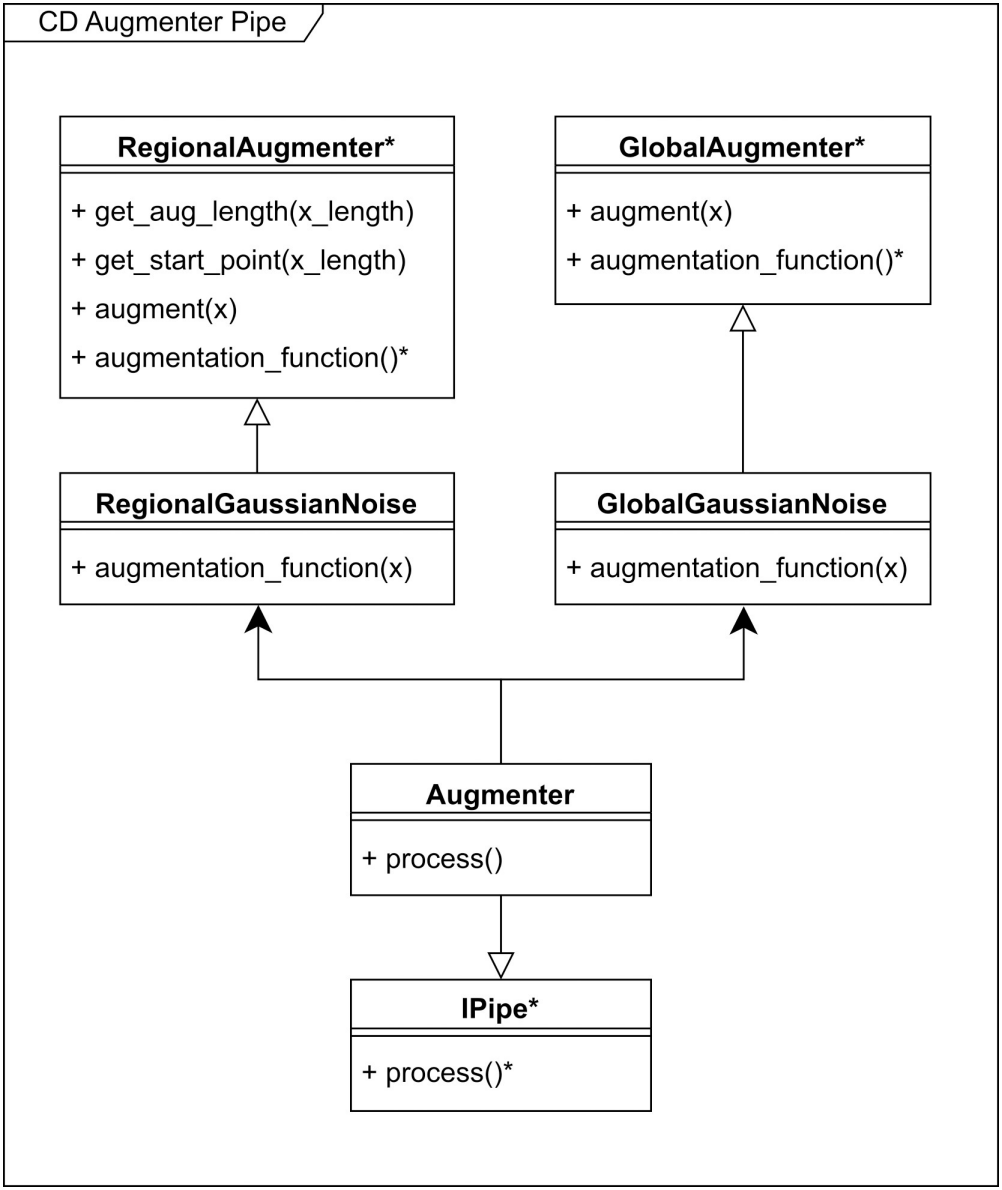
Class diagram depicting the design of the data augmentation. Abstract classes, abstract functions and abstract properties are shown with *.

As can be seen in Figs 3 and 4, we have also implemented the data serving portion flexibly, such that different schemes for data drawing and data augmentation can be incorporated. Data serving in general, is extendable with custom Pipe implementations of the IPipe interface as seen on Fig 3. Similarly, the Augmenter pipe, shown in Fig 4, is extendable with specific implementations of the Regional- or GlobalAugmenter, depending on augmentation requirements. We believe that the current implementation covers the majority of the studies currently conducted in this field. Both the random sampling and augmentation design is inspired by Perslev et al. 2021 [5] but has been adopted and altered for our own pipeline design.

Class diagram depicting the data serving part of the pipeline using a generic Pipe class. Abstract classes, abstract functions and abstract properties are shown with *.

### Demonstration

To demonstrate the Common Sleep Data Pipeline, we downloaded and preprocessed all data sets described in Table 1, resulting in 21 HDF5 files of a combined size of 3719 GB. As previously mentioned, we then used this data to train two quite different sleep scoring models, U-Sleep [5] and L-SeqSleepNet [10]. Both models used the random sampler for training, but a deterministic sampler for validation and testing. Furthermore, they also used the same implementation of data augmentation.

However, U-Sleep takes an input which matches the format of the standardized data and therefore needs no further preprocessing in order to train and test, while L-SeqSleepNet takes an input of spectrograms based on data with a sample rate of 100 Hz. Therefore, two extra steps of processing were introduced for L-SeqSleepNet: resampling and conversion into spectrograms. Data was split such that 8 smaller data sets (labelled “hold-out” in Table 1) were kept apart as testing data. The remaining 13 datasets (labelled “training” in Table 1) were split in train, validation and test partitions, with the validation and test partitions taking up 15% each. The models were then trained and validated on all the training and validation partitions simultaneously. Afterwards the models were evaluated on the “hold-out” datasets together with the test partitions from the “training” datasets. This allows investigating both within- and across-cohort generalization.

**Fig 5:**
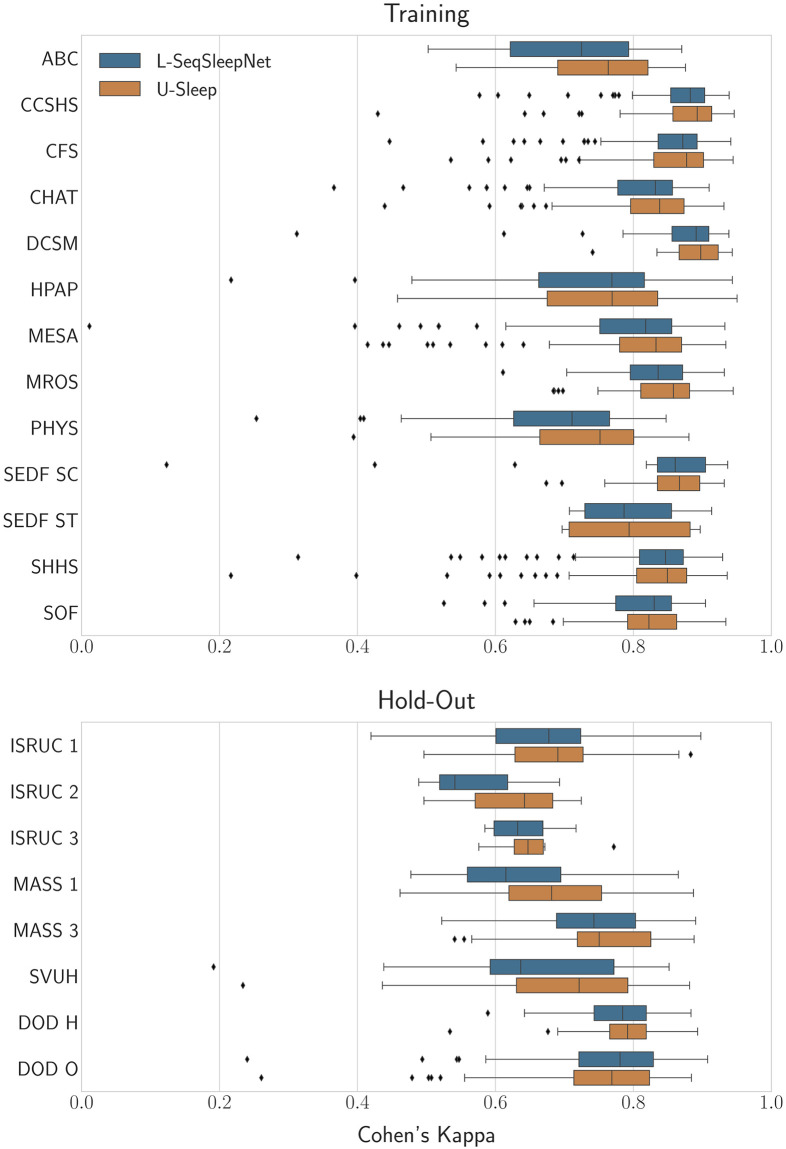
Distribution of per-recording kappa scores achieved on all data sets. The upper plot shows scores for the training set, while the lower plot shows scores for the hold-out sets.

Fig 5 shows a distribution of kappa values for both models for the test partitions of all data sets, where a single value refers to the kappa for one record. The model predictions used to create this plot can be found in S3 and S4 Files. For the purpose of the demonstration, a more aggressive training strategy was employed compared to Perslev et al. [5]; we chose a higher learning rate on a decaying schedule and a shorter training duration. Still, training the U-Sleep model with our pipeline reached a mean F1 performance score fairly close to their results, as can be seen in the supporting information S1 Table.

## Discussion

The design choices made in this implementation naturally leave room for specializations and extensions. We believe that a shared framework like the CSDP presents a lot of benefits for the scientific community, increasing reproducibility and decreasing the barrier of entry. In particular, the modular aspect of this design should lower the development time needed when new data sets are added to the pool.

A very practical limitation of the data preprocessing step as it is currently implemented, is that the download of raw data is only implemented for the datasets originating from https://sleepdata.org/. Documentation for downloading the other datasets can be found in the repository, but in the future, all datasets will be available for automatic download. We also hope to extend the number of data sets which already have preprocessing modules implemented.

Another capability which we intend to include is the ability to include more features from the source data. For now the focus has been EEG and EOG, since those signals are used to train U-Sleep and L-SeqSleepNet. However, we acknowledge that for the pipeline to be of even greater use to the scientific community, other variables need to be supported (for example ECG, EMG and heart-rate). Fortunately this would only require a light extension of the current design in a future version.

It is of course also possible to include information that is not in a time series format (such as gender, BMI, ethnicity, anamnesis). However, we expect that such information may need to be served in different ways depending on the specific type of information, and thus expect that it will be development on a case-by-case basis. Should anyone in the community be interested in this direction, we would be very happy to discuss it.

Concerning the data serving portion of the pipeline, the default behavior in the present version is randomly sampling in batches, to overcome memory constraints. However, it is not guaranteed that future uses of CSDP include big data—meaning that relevant extensions could include customized data loading, possibly more compute efficient single loading of everything if it fits in memory.

It’s also possible, particularly when considering different types of data augmentation, that a user would prefer a different ‘savepoint’ in the pipeline, for instance using pre-computed spectrograms. While that is certainly possible to do in the current implementation, future versions could make it easier to define this in a rigorous manner.

Related to this, if a user wanted to only use the preprocessing portion without a machine learning ‘head’, or with a head that was radically different from what we have designed for, it is already possible to only use the first part of the pipeline. This is reflected both in the structure of the repository as well as the user guide.

## Supporting information

S1 File Memory peaks during file-format analysis. The individual values, mean and standard deviation for file-format memory peaks during the analysis.
(XLSX)

S2 File Load times during file-format analysis. The individual values, mean and standard deviation for file-format load times during the analysis.
(XLSX)

S3 File L-SeqSleepNet predictions. Sleep-stage predictions for L-SeqSleepNet on each individual session from the test-partitions, together with the per-record calculated kappa score.
(XLSX)

S4 File U-Sleep predictions. Sleep-stage predictions for U-Sleep on each individual session from the test-partitions, together with the per-record calculated kappa score.
(XLSX)

S1 Table Per-dataset F1 scores. Per-dataset F1 scores for our U-Sleep model predictions. These have been calculated using the supporting information from S4 File. These scores are compared against the U-Sleep results reported by Perslev et al 2021.
(PDF)
