# Supplementary material for: Common sleep data pipeline for combined data sets
Source: PLoS One. 2024 Aug 6;19(8):e0307202. doi: 10.1371/journal.pone.0307202 (PMC11302895; doi:10.1371/journal.pone.0307202)
Supplement: S1 Table — Per-dataset F1 scores for our U-Sleep model predictions. These have been calculated using the supporting information from S4 File. These scores are compared against the U-Sleep results reported by Perslev et al 2021. (PDF) [file pone.0307202.s005.pdf]

Table 1. The global F1 mean scores for each individual dataset, achieved by the U-Sleep model in our pipeline (CSDP) and the pipeline used in Perslev et al. 2021. Our results are calculated from the U-Sleep predictions also found in the supplementary information (S4 File). The Perslev et al. results are reported directly from the 'mean' column in table 2 of their article.

Dataset	Model	Global Mean F1
ABC	CSDP	0.79
	Perslev et al.	0.77
CCSHS	CSDP	0.85
	Perslev et al.	0.85
CFS	CSDP	0.82
	Perslev et al.	0.82
CHAT	CSDP	0.83
	Perslev et al.	0.85
DCSM	CSDP	0.84
	Perslev et al.	0.81
HPAP	CSDP	0.78
	Perslev et al.	0.78
MESA	CSDP	0.79
	Perslev et al.	0.79
MROS	CSDP	0.78
	Perslev et al.	0.77
PHYS	CSDP	0.79
	Perslev et al.	0.79
SEDF-SC	CSDP	0.80
	Perslev et al.	0.79
SEDF-ST	CSDP	0.83
	Perslev et al.	0.76
SHHS	CSDP	0.81
	Perslev et al.	0.80
SOF	CSDP	0.78
	Perslev et al.	0.78
ISRUC-SG1	CSDP	0.74
	Perslev et al.	0.77
ISRUC-SG2	CSDP	0.70
	Perslev et al.	0.76
ISRUC-SG3	CSDP	0.73
	Perslev et al.	0.77
MASS-C1	CSDP	0.71
	Perslev et al.	0.73
MASS-C3	CSDP	0.79
	Perslev et al.	0.80
SVUH	CSDP	0.74
	Perslev et al.	0.73
DOD-H	CSDP	0.82
	Perslev et al.	0.82
DOD-O	CSDP	0.77
	Perslev et al.	0.79
